# Biosynthesis of Levan, a Bacterial Extracellular Polysaccharide, in the Yeast *Saccharomyces cerevisiae*

**DOI:** 10.1371/journal.pone.0077499

**Published:** 2013-10-11

**Authors:** Jaco Franken, Bianca A. Brandt, Siew L. Tai, Florian F. Bauer

**Affiliations:** Institute for Wine Biotechnology, Stellenbosch University, Stellenbosch, Western Cape, South Africa; Auburn University, United States of America

## Abstract

Levans are fructose polymers synthesized by a broad range of micro-organisms and a limited number of plant species as non-structural storage carbohydrates. In microbes, these polymers contribute to the formation of the extracellular polysaccharide (EPS) matrix and play a role in microbial biofilm formation. Levans belong to a larger group of commercially important polymers, referred to as fructans, which are used as a source of prebiotic fibre. For levan, specifically, this market remains untapped, since no viable production strategy has been established. Synthesis of levan is catalysed by a group of enzymes, referred to as levansucrases, using sucrose as substrate. Heterologous expression of levansucrases has been notoriously difficult to achieve in *Saccharomyces cerevisiae*. As a strategy, this study used an invertase (Δ*suc2*) null mutant and two separate, engineered, sucrose accumulating yeast strains as hosts for the expression of the levansucrase M1FT, previously cloned from *Leuconostoc mesenteroides*. Intracellular sucrose accumulation was achieved either by expression of a sucrose synthase (Susy) from potato or the spinach sucrose transporter (SUT). The data indicate that in both Δ*suc2* and the sucrose accumulating strains, the M1FT was able to catalyse fructose polymerisation. In the absence of the predicted M1FT secretion signal, intracellular levan accumulation was significantly enhanced for both sucrose accumulation strains, when grown on minimal media. Interestingly, co-expression of M1FT and SUT resulted in hyper-production and extracellular build-up of levan when grown in rich medium containing sucrose. This study presents the first report of levan production in *S. cerevisiae* and opens potential avenues for the production of levan using this well established industrial microbe. Furthermore, the work provides interesting perspectives when considering the heterologous expression of sugar polymerizing enzymes in yeast.

## Introduction

Fructans are fructose polymers consisting of multiple fructose units, which occur in a broad range of micro-organisms and a limited number of plant species as non-structural storage carbohydrates [1]. These molecules have been increasingly used in production of functional foods and pharmaceutical formulations due to their pre-biotic properties and other health enhancing roles [2]. Three main molecules group under the broad definition of fructans, namely levan, inulin and small chain fructo-oligosaccharides (scFOS). The key distinction between these polymers is found in the linkage type between individual fructose units. Characteristically, inulin type fructans have β(2 → 1) and levan type fructan have β(2 → 6) bonds [3]. Microbial inulin has been isolated with degrees of polymerization ranging between 20 and 10 000, whereas levan polymers generally have a DP > 100. Small chain fructo-oligosaccharides usually have a DP of less than 9. Sucrose acts as the fructose donor for all fructan polymers. The primary sucrose acts as a fructose acceptor to start chain lengthening, leaving a glucose moiety remaining at one end of the polymer. The biological function of fructan polymers in microbes are considered to be providing a physical barrier, enhancing resistance to environmental stresses and aiding in nutrient assimilation. Through these combined structural and functional contributions, fructans also play a determining factor in the pathogenesis of disease related microorganisms [3]. In addition, it has recently been shown that levan, although not essential, does form part of extracellular matrix of *Bacillus subtilis* cultures grown in sucrose rich media. In this setting, the polymer contributed to biofilm robustness and was proposed to act as a nutrient reserve [4].

Fructan polymers are synthesized by a group of enzymes referred to as fructosyltransferases (FTFs). The FTFs are characteristically able to catalyse two different reactions: (i) trans-glycosylation, using the growing fructan chain (in the case of polymerization), sucrose, or gluco- and fructosaccharides (in the case oligosaccharide synthesis) as the acceptor substrate; (ii) and hydrolysis of sucrose, using water as the acceptor [3]. FTFs have been cloned from a variety of evolutionary diverse bacterial species and several functional domains have been identified in this group of proteins. The first is an N-terminal signal peptide, second is a catalytic domain of ~ 500 amino acids, and finally, in the case of some Gram-positive bacteria, a cell-wall binding domain has been designated. The N- and C-terminal regions of the FTFs contain amino acid stretches of variable lengths. 

The commercial potential of fructans motivated various research programmes to investigate the feasibility of using heterologous expression systems for the production of fructosyltransferases or their associated products. Fructosyltransferases that catalyse the production of scFOS, for instance, have been functionally expressed in *S. cerevisiae* and other eukaryotic expression systems [5]. Several studies have described varying degrees of success with the expression of levansucrases in plants [1,6,7]. When expressing fructosyltransferases in plants, the aim was generally to accumulate the specified polymer in a target organ or tissue and not to have the enzymes secreted. In contrast to plant studies, microbial expression systems generally aimed to have the enzymes secreted to the media where the substrate, sucrose, would be abundant and could be used for fructan production. Limited successes have, however, been reported when using microbial systems expressing levansucrases. The rare exceptions include, sacB, the extracellular levansucrase from *Bacillus subtilis*, which was expressed in *S. cerevisiae*, but shown to remain intracellular in its precursor form [8]. Furthermore, functional expression and secretion of lsdA, cloned from *Gluconacetobacter diazotrophicus*, was achieved in *Pichia pastoris*. However, the recombinant enzyme only produced low molecular weight levan [9]. M1FT from, *Leuconostoc mesenteroids*, was also successfully expressed and secreted in *P. pastoris*, with yields suggesting industrial viability [10]. The M1FT gene was also expressed in *Escherichia coli*, with large amounts of the enzyme being retained as cytoplasmic inclusion bodies. 

**Figure 1:**
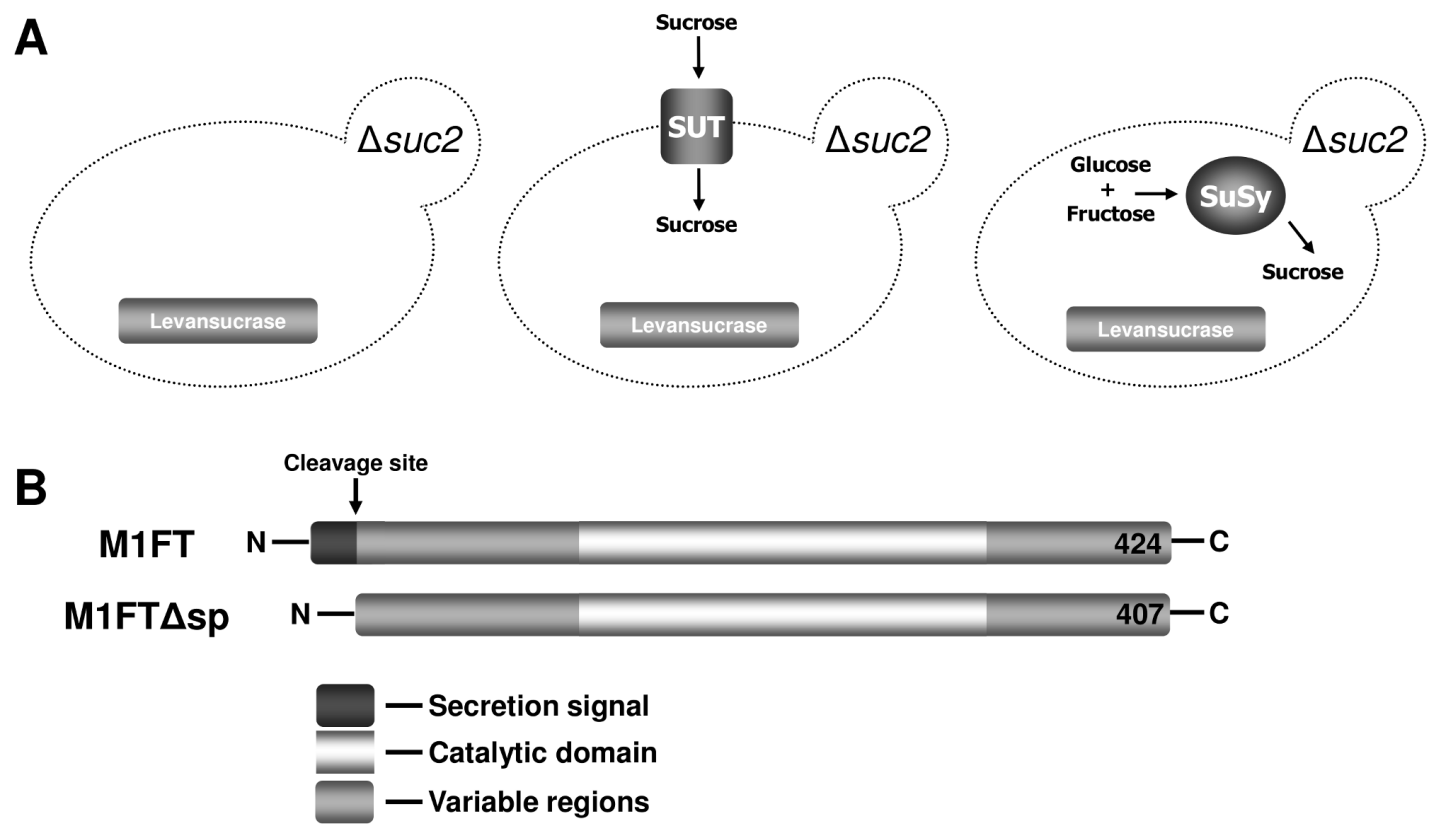
Diagrammatic representation of experimental layout and levansucrases used in this study. (A) Yeast strains used to express the levansucrase M1FT; BY4742Δ*suc2* and the two sucrose accumulating strains BY4742Δ*suc2*-SuSy and BY4742Δ*suc2*-SUT. (B) M1FT, a levansucrase from *Leuconostoc mesenteroids* and also a truncated version of M1FT, without its predicted secretion signal, was expressed in the different yeast strains.

Taken together, the previous reports indicate that choice of enzyme, cellular location, substrate concentration and also the presence of signal sequences are factors that contribute to the successful heterologous expression of levansucrases. In order to create varying scenarios, investigating the possibility of either of intra- or extracellular enzymatic activity, an invertase null mutant (Δ*suc2*) was used as a base strain to generate two separate sucrose accumulating *S. cerevisiae* strains (Figure 1). These strains where used for the expression of M1FT. The sucrose accumulation strains where constructed by introducing either a potato sucrose synthase [11], referred to as SuSy, or by growing strains expressing the spinach sucrose transporter referred, to as SUT [12]. The two sucrose accumulation strategies are also in line with common conditions in which *S. cerevisiae* is applied in industrial contexts. In wine fermentations, glucose and fructose make up the available sugars in the grape must, presenting the substrates used by SuSy for sucrose synthesis. While sucrose is a dominant sugar present in industrial media that favour biomass accumulation, such as molasses, and would function as the target of SUT import. 

The data indicate that the M1FT constructs result in levan polymer production when expressed in the sucrose accumulating *S. cerevisiae* strains. Furthermore, removal of the predicted M1FT signal sequence results in accumulation of levan inside the cell. Depending on media conditions, levan production in the SUT strains could be escalated to high levels that accumulated within the growth medium and also within the modified cells. In short, this work provides the first report of levan producing strains of the yeast *S. cerevisiae* and lays the foundation for the future exploration of this yeast as a vector for sugar polymer production in various contexts.

## Materials and Methods

### Strains, plasmids and culture conditions

*Escherichia coli* DH5α (GIBCO-BRL/Life Technologies) was used as host for the cloning and propagation of all plasmids. Plasmid-bearing *E. coli* strains were grown at 37°C on Luria-Bertani (LB) medium [13], supplemented with ampicillin for selection. General procedures for cloning, DNA manipulations, transformations and agarose gel electrophoresis were performed using standard techniques [13]. Restriction enzymes, T4 DNA-Ligase, and Expand Hi-Fidelity polymerase used in the enzymatic manipulation of DNA were obtained from Roche Diagnostics (Randburg, South Africa) and used according to the specifications of the supplier. All yeast strains used in this study are derived from the BY4742 (S288c) genetic background and are listed in Table 1. Yeast strains were grown either on rich YPD (1% yeast extract, 2% peptone, 2% glucose) or on minimal SCD media, containing 0.67% (w/v) yeast nitrogen base (YNB) without amino acids (DIFCO) and 2% (w/v) glucose, supplemented with amino acids according to the specific requirements of the respective strains. For sucrose accumulation and levan production experiments, cultures were grown in SCD media containing either 4% (w/v) glucose and 4% (w/v) fructose (SCDF) for the sucrose synthase (SuSy) expressing strains or with 5% (w/v) sucrose and 3% (w/v) glucose (SCDS) in the case of the sucrose transporter (SUT) carrying strains. Similarly, for experiments using rich media, strains were grown in 1% yeast extract, 2% peptone and either 4% (w/v) glucose and 4% (w/v) fructose (YPDF) for the sucrose synthase (SuSy) bearing strains or with 5% (w/v) sucrose and 3% (w/v) glucose (YPDS) for the SUT bearing strains. Molasses media (YE-Molasses) was prepared by diluting molasses to the same glucose fructose and sucrose concentrations as YPDS and supplementing with 1% yeast extract as nitrogen source. 

**Table 1:** Yeast strains used in this study.

**Strain**	**Phenotype**	**Reference**
BY4742	*MATα leu2 lys2 his3 ura3*	Euroscarf
BY4742∆*suc2*	*MATα leu2 lys2 his3 ura3 suc2::LEU2*	This Study
BY4742∆*suc2*-SuSy	*MATα leu2 lys2 his3 ura3 suc2::LEU2 spr3::*SuSy	This Study
BY4742∆*suc2*-SUT	*MATα leu2 lys2 his3 ura3 suc2::LEU2 spr3::*SUT	This Study

### Construction of SUT and SUSY integration cassettes

All primers and plasmids used in this study are indicated in Tables 2 and 3 respectively. The SuSy open reading frame (ORF) was amplified from potato cDNA according to the originally cloned sequence [11]. The primer pair SuSy-F and SuSy-R was used to amplify the ORF, which was cloned into the cloning vector pGEM-Teasy (Promega). The cloned ORF was subcloned into the *PGK1*_promoter_ (*PGK1*_p_)-*PGK1*_terminator_ (*PGK1*_t_) cassette of the yeast expression vector pHVXII, using restriction sites introduced through PCR (*Bam*HI and *Bgl*II in the 5’ and 3’ regions of the amplified SuSy ORF). The SuSy integration cassette was constructed by combining the expression cassette *PGK1*_p-_SuSy-*PGK1*_t_ with the *HIS3* auxotrophic marker and flanking regions for integration into the *SPR3* locus using a fusion PCR strategy. The *PGK1*_p-_SuSy-*PGK1*_t_ cassette was amplified from pHVXII-SuSy plasmid using the primer pair *PGK1*-LacZ-F and *PGK1*t-*SPR3*-R. The *HIS3* gene was amplified as an auxotrophic marker was from YDp-H [14] using the primers *SPR3*-*HIS3*F and *HIS3*-*PGK1*p-R. PCR products were ligated into pGEM-Teasy vector and sequenced using an ABI PRISM^TM^ automated sequencer to confirm the integrity of the amplicons. Positive SuSy clones were digested using *Apa*I and *Sbf*I and *HIS3* clones were digested with *Apa*I and *Sal*I. SuSy and the *HIS3* auxotrophic marker were then ligated and the resulting product was used as template for a fusion PCR reaction. The resulting *SPR3*-HIS3-*PGK1*_p_-SUSY-*PGK1*_t_-*SPR3* cassette was transformed using the lithium acetate transformation protocol [15] into the *S. cerevisiae* strains BY4742∆*suc*2::*LEU2*. The ∆*suc*2::*LEU2* disruption cassette in this strain was amplified using the primers *SUC2*-KO-Fw-*LEU2*p and *SUC2*-KO-Rv-*LEU2*t using pHVXII as template. This primer par amplifies the *LEU2* gene together with flanking regions for integration at the *SUC2* locus. The sucrose transporter gene was provided by the Institute for Plant Biotechnology (Stellenbosch University, South Africa) as an expression construct in the yeast vector pPVD1. The ORF was cloned into the *PGK1*p-*CYC3*t expression cassette, neighbouring the *HIS3* auxotrophic marker. The primers *SPR3*-FW-*PGK1*p and *SPR3*-RV-HIS3p, were used to amplify the entire *PGK1*_p_-SUT-*CYC3*_t_ expression cassette and also the *HIS3* auxotrophic marker. The fragment was directly used as an integration cassette. The respective integration cassettes were transformed into the BY4742∆*suc*2::*LEU2* strain to yield the strains BY4742∆*suc*2-SUT and BY4742∆*suc*2-SuSy. Successful integration was confirmed using primers that bind within the integration cassette paired with primers specific to the up and downstream regions in the yeast genome. The *SPR3* locus for both integrations were amplified and sequenced to confirm the integrity of the cassettes.

**Table 2:** Primers used in this study.

**Primer**	**Sequence**
SuSy-F	5’-GATCGGATCCATGGCTGAACGTGTTTTGACTCGTG-3’
SuSy-R	5’-GATCAGATCTTCACTCAGCAGCCAATGGAACAG-3’
M1FTc-EcoR1-F	5’- GATCGAATTCATGAAAAGCACCCCTGAGAA-3’
M1FTt-EcoR1-F	5’- GATCGAATTCATGTGGACCCGCGCCGATG-3’
M1FT-Xho1-R	5’- CCTGCTCGAGTTACTTGAGCGTGACGTCG-3’
*SUC2*-KO-Fw-*LEU2*p	5’-tttataacctctattttacttcccttacttggaacgtgaccacgttggtcaagatcaca-3’
*SUC2*-KO-Rv-*LEU2*t	5’-cactaacgtatatgatgcttttgcaagctttccttagcaaggattttcttaacttcttcg-3’
*SPR3*-FW-*PGK1*p	5’-AAAAGGGAGTCGGTTGTCAACAGACTGTCCTGTCGAATTTCCCAAG GATCCGTGGCCTCTTATCGAG-3’
*SPR3*-RV-*HIS3*p	5’-AGCACTATCTGTGGAATGGCTGTTGGAACTTTTTCCGATTACTGAGA GTGCACCATAAATTCCCG-3’
*SPR3*-*HIS3*-F	5’-AAAAGGGAGTCGGTTGTCAACAGACTGTCCTGTCGAATTTCCCAA CTGAGAGTGCACCATAAATTCCCGT-3’
HIS3-*PGK1*_P_-R	5’-CTGAACGAGGCGCGCTTTCCTTTTTTCTTTTTGCTTTTTCTTTTTTTTA GCTTTCTAACTGATCTATCCAAAAC-3’
*PGK1*t-*SPR3-*R	5’-AAAATTCGCTCCTCTTTTAATGCCTAATCGGAAAAAGTTCCAACAGCC ATTCCACAGATAGTGCT-3’
*PGK1*-LacZ-F	5’-GATCCTCGAGAGCTTTCTAACTGATCTATCCAAAACT-3’

Underlined sequences indicate introduced restriction sites.

**Table 3:** Description of the plasmids used in this study.

**Plasmid**	**Genotype**	**Reference**
YCplac33	CEN4 *URA3*	17
YCplac33-*PGK1*pt	CEN4 *URA3 PGK1p PGK1*_*t*_	This study
YCplac33-*PGK1*-M1FT∆sp	CEN4 *URA3 PGK1*_*p*_ -M1FT∆sp –*PGK1*_*T*_	This study
YCplac33-*PGK1*-M1FT	CEN4 *URA3 PGK1*_*p*_ -M1FT–*PGK1*_*t*_	This study
pHVXII	2µ *LEU2 PGK1*_pt_	16
pHVXII-SUSY	2µ *LEU2 PGK1*_*p*_ -SuSy –*PGK1*_*t*_	This study
pBluescript-M1FT	M1FT	IPB
pPVD1-S21	2µ *HIS3 PGK1*_p_-soSUT1-*CYC3*_t_	IPB

### Construction of M1FT expression vectors

The yeast vector YCplac33-*PGK1*_*pt*_ was used for the over-expression of the M1FT gene and also the truncated version, M1FT∆sp. This construct was created by ligating the *Bam*HI/*Nar*I excised *PGK1*promoter/*PGK1*terminator cassette from pHVXII [16] into YCplac33 [17] digested with the same enzymes. The M1FT and truncated M1FT∆sp genes were amplified from the pBluescript-M1FT plasmid (provided by the Institute for Plant Biotechnology, Stellenbosch University) using the either one of the forward primers M1FTc-EcoR1-F (for M1FT) or M1FTt-EcoR1-F (for M1FT∆sp) in combination with M1FT-Xho1-R as reverse primer. M1FT∆sp was constructed with a truncation of 51bp to remove the signal peptide predicted by SignalP 3.0 software [18]. PCR products were ligated into pGEM-T Easy vector (Promega) and. Positive clones were digested with *Eco*R1 and *Xho*1 (which were introduced by PCR) and ligated into *PGK1*pt promoter/terminator cassette in the YCplac33-*PGK1*pt plasmid with resulting in the generation of YCplac33-*PGK1*pt-M1FT and YCplac33-*PGK1*pt-M1FT∆sp yeast expression vectors. Final constructs where sequenced using an ABI PRISM^TM^ automated sequencer to rule out mutations derived during PCR amplification.

### Sugar extraction and analysis by thin-layer chromatography

Overnight cultures of strains were inoculated into appropriate 100mL culture media and grown for 2 days at 30°C to saturation. Cells were harvested from 100mL cultures by centrifugation at 5000 rpm for 2 min. The harvested cells were resuspended in 1mL distilled H_2_O and transferred to 2mL centrifugation tubes. Cells were washed with 1mL MiliQ water and resuspended in 500µL MiliQ. Addition of ~300µL glass beads to suspension was followed by vortexing at top speed for 10min. Cell suspensions where cleared by centrifugation at 12000rpm for 30 seconds. The aqueous cell extract was transferred to 1.5mL centrifugation tubes and dried at 55°C. Dried extracts where resuspended in 20µL MiliQ H_2_O (20x concentration) and stored at 4°C. Samples (2uL) were spotted on thin layer chromatography (TLC) Silica gel 60 F_254_ foils (Merck), and separated using a mobile phase of butanol:acetic acid:water (50:30:15). Standards were made up as mixture of fructose, sucrose and levan (*Zymomonas mobilis, Fluka* Biochemika) at a concentration of 10g/L each. Fructose containing sugars were specifically stained with a urea spray [19], and developed at 110°C until the stained bands could be clearly visualized. For the analysis of levan in the growth medium, 1 µL of media was either spotted directly on the TLC plates (for BY4742Δ*suc2*-SUT-M1FT grown in YPDS) or levan was precipitated from 10 mL of media using ethanol and concentrated in 20 µL of (500X) and 1 µL used for spotting on TLC plates. Sugar extracts where collected from biological triplicates in an experimental set and experimental sets where repeated at least once for confirmation. 

### Quantification of levan produced

Levan quantification was performed as described previously [1]. Samples (1µL) were analysed using TLC, in combination with a concentration series of 0.625, 1.25, 2.5, 5, 10, 20, 30 µg of levan from *Zymomonas mobilis* (Sigma). The stained fructans were photographed using a G-Box, SynGene gel camera combined with GeneSnap software v. 7.09.11 (SynGene). A densitometric analysis was done on the coloured levan spots on the TLC using the Genetools v.4.01.04 (Synoptics Ltd) software package. The concentration of the spotted levan samples was then calculated using the standard curve generated from the concentration series. Levan concentration was standardised against total protein concentration of the corresponding cultures or used to calculate total levan produced per litre of media. Total protein quantification was done at Stellenbosch University’s Central Analytical Facilities (CAF) using 2-D Quant Kit (GE Healthcare). Biological triplicates where used for the determination of levan concentration. Standard deviation (SD) calculated between the sample repeats is provided as absolute values. Differences between assay values were calculated at a p < 0.05 level using the student paired t-test.

### Levan polymer purification and characterisation

Cultures of BY4742∆*suc2*-SUT-M1FT∆sp where grown in 1L of growth medium containing glucose and sucrose to saturation over 48 hours. Cells were harvested by centrifugation at 5000 rpm for 2 min. Levan was subsequently extracted as described above. The cell extract was treated with DNAse and RNAse and incubated at 37°C for 1 hour. Samples were subsequently incubated with Proteinase K (20mg/L) at 37°C for 1hour. Samples were then centrifuged at 10 000 rpm at 4°C for 10 min and the supernatant aspirated into new 1.5 mL eppi. Ice cold 100% ethanol was then added to samples to make up 1.5 mL and polymer was the precipitated overnight at -20°C. Levan was collected by centrifugation at 12 000rpm for 10 min at 4°C. The supernatant was dispensed in a new tube and the pellet was dried in a 55°C oven for 40 min followed by 5 min drying in a vacuum centrifuge (SpeedyVac). Approximately 10mg of purified and dried polymer was dissolved in 1 mL deuterium monoxide (D2O) with the addition an external standard tetramethylsilane (TMS) to a final concentration of 4.5 mM. Samples were placed in NMR tubes and analysed at the Central Analytical facility (CAF) at Stellenbosch University. A 600MHZ Varian INOVA NMWR was used to create the proton (^1^H) spectra of the samples. The linkage type of the polymer was analyzed by ^13^C-NMR spectrometry. Chemical shifts were measured (and reported in parts per million) downfield from TMS. Peak assignment was based on the report of Shimamura and co-workers [20]. As a control, a commercially available levan from *Zymomonas mobilis* was analysed in parallel (Fluka Biochemika).

The degree of polymerization of the purified polymer was expressed as a ratio of glucose *vs* fructose after polymer hydrolysis. Purified polymer where hydrolysed in 2M trifluoroacetic acid at 100°C for 2h. Complete polymer hydrolysis was confirmed using TLC. Glucose and fructose were quantified on an Arena 20XT enzyme robot using the recommended kits (Thermo Fisher Scientific). 

### Alcoholic fermentation of M1FT strains

Fermentations were done in triplicate in either MS300 [21] synthetic wine media or YPDF (both media with 50g/L glucose and 50g/L fructose) at 30°C. Strains were inoculated into media to OD_600_ of 0.1 from respective overnight cultures. Fermentation progress was monitored as weight loss (CO_2_ loss) and throughout the course of the fermentations. After completion of fermentation, cells were harvested by centrifugation at and cell extract were prepared for analysis of sucrose and levan production. The supernatant was used to determine the amount of residual sugars (glucose and fructose) at the end of fermentation and also the ethanol concentration. Ethanol quantification by HPLC was done as described previously [22]. Glucose and fructose concentration was determined using an Arena 20XT enzyme robot using the recommended kits (Thermo Fisher Scientific). Biological triplicates were used for the fermentation experiments.

## Results

### Accumulation of sucrose in *S. cerevisiae* and subsequent levan production by M1FT in modified yeast strains

**Figure 2:**
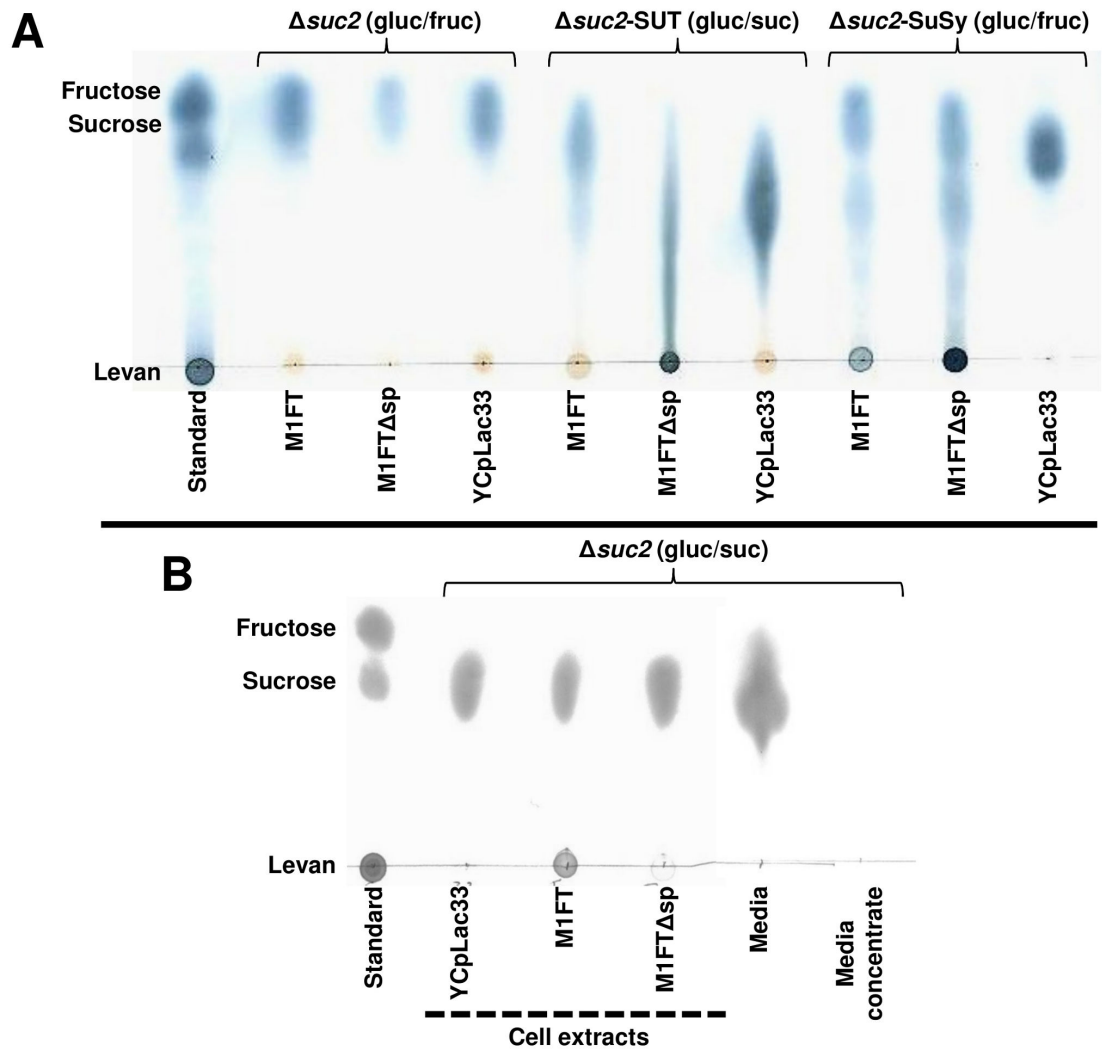
Thin-layer chromatographic analysis of levan production by M1FT. The presented TLC plates are representative of observations confirmed by at least three biological repeats. Fructose containing molecules were visualized using a urea spray after separation on TLC plates. (A) Cell extracts of strains expressing either the full-length M1FT, M1FT without signal sequence (M1FTΔsp) or the vector (YCpLac33) as control in BY4742Δ*suc2* (grown in glucose and fructose containing media) and the two sucrose accumulating strains BY4742Δ*suc2*-SUT and BY4742Δ*suc2*-SuSy (grown in glucose and sucrose; and glucose and fructose containing media respectively). (B) Levan production by M1FT in BY4742Δ*suc2* grown in media containing glucose as carbon source, supplemented with 5% sucrose. Cell extracts are indicated with a dashed line. The growth medium was either spotted directly from the supernatant (Media) or as a 500X concentrate of the same supernatant after ethanol precipitation (Media concentrate); in both cases, no levan production could be detected.

To ensure intracellular accumulation of sucrose and also avoid fructose polymer degradation by the native yeast invertase, a Δ*suc2* genetic background was used for all expression studies. Two strategies where pursued to generate yeast strains that are able to accumulate high intracellular levels of sucrose, that can subsequently be used as substrate by the levansucrase, M1FT, for levan production. The first approach makes use of the sucrose synthase (SuSy) from potato [11] and the second uses expression of the spinach sucrose transporter, soSUT (referred to as SUT in this study [12]). Both these genes where integrated into the BY4742Δ*suc2* genome under the expression of the *PGK1* promoter and terminator. Since SuSy uses glucose and fructose as substrates for sucrose synthesis, the SuSy trains where grown in media supplemented with glucose and fructose as carbon sources (Figure 2A). Similarly, the sucrose transporter strains where grown in glucose media, supplemented with sucrose, which can subsequently be imported into the SUT carrying cells (Figure 2A). In addition, the BY4742Δ*suc2* strains expressing M1FT where grown in both glucose and fructose (Figure 2A) and also glucose media containing sucrose (Figure 2B) to act as control strain in both media conditions. Extracts from the cultures where evaluated using TLC to assess the ability of the strains to accumulate fructose, sucrose and polymers containing fructose. Unsurprisingly, only fructose was detected in BY4742∆*suc2* control strains grown in the presence of glucose and fructose (Figure 2A). Sucrose was, however, detectable in this strain when grown in the presence of sucrose (Figure 2B). In the absence of *SUC2* this is expected, since it is known that sucrose can be taken up by cells of *S. cerevisiae* through the Agt1p disaccharide transporter [23]. Furthermore, the data confirm that both BY4742∆*suc2*-SuSy and BY4742∆*suc2*-SUT (indicated by the YCpLac33 line for each strain) are able to accumulate sucrose inside the yeast cells (Figure 2A). Interestingly, the two sucrose accumulation strains do not accumulate sucrose to the same level. Expression of SuSy results in a higher build–up of sucrose compared to the sucrose transporter carrying strain. Furthermore, extracts from BY4742∆*suc2*-SUT grown in sucrose containing media consistently resulted in TLC patterns that suggest the presence of fructose containing molecules that migrate slower than sucrose. However, the combined concentration of these fructose containing molecules appear to be notably higher than the sucrose levels observed in the BY4742∆*suc2* strain when grown in the presence of 5 % sucrose. It is not currently clear to what these unexpected observations regarding the SUT transporter can be attributed. 

As expected, M1FT expression in the BY4742∆*suc2* background did not result in any levan production when the cultures were grown in media containing glucose and fructose (where no sucrose is present). Levan production was, however, detected in association with the cells when the cultures were grown in SCDS media (minimal media containing sucrose and glucose). In the same conditions, no polymer production was detectable in the growth medium even when the growth medium was precipitated with ethanol to concentrate levan that may have been present (Figure 2B; Media and Media concentrate). A likely explanation for this observation could be that, unlike small carbohydrates, fructans form specific, non-covalent interactions with biological membranes [24]. This would, at lower levan concentrations, sequester the polymer from the media. As with many levansucrases, M1FT has a distinguishable secretion signal sequence in the N-terminal region of the protein. Deletion of the predicted signal sequence resulted in a marked decrease in levan production in the BY4742∆*suc2* background, when grown in the presence of sucrose (Figure 2B). This indicates that levan production is predominantly not intracellular in this specific strain. In both the sucrose accumulation strains levan production was detectable at low levels when expressing the full-length M1FT (Figure 2A). However, production by M1FT in the SuSy background was visibly higher than in the SUT background. Removal of the predicted signal sequence (M1FT∆sp) resulted in a drastic increase of intracellular levan in both the sucrose accumulation strains (Figure 2A; BY4742∆*suc2*-SuSy and BY4742∆*suc2*-SUT in combination with M1FT∆sp). Even though sucrose is extractable from the BY4742∆*suc2* cells when grown in the presence of sucrose (YCpLac33 line in Figure 2B), it is appears less than both accumulation strains and also does not support intracellular levan synthesis (by M1FT∆sp) to nearly the same extent as the SUT and SuSy bearing strains. The BY4742∆*suc2-*SuSy-M1FT∆sp strains produced an average 0.136mg levan/mg protein (SD 0.078 mg levan/mg protein) and BY4742∆*suc2-*SUT-M1FT∆sp strains 0.178mg levan/mg protein (SD 0.061mg levan/mg protein). The difference between the two strains was, however, not statistically significant (p ˃ 0.05). 

Interestingly, streaking can be observed on the TLCs of levan producing strains in both the sucrose accumulation strains (Figure 2A). The streaks occur slightly below sucrose, which could indicate that levan type fructans of various degrees of polymerisation (DP) are being formed. M1FT was previously shown to produce fructooligosaccharides (FOS), specifically 1-kestose, nystose and 1,1,1-kestopentaose in addition to levan [25]. The production of FOS by M1FT was shown to increase with increasing sucrose concentrations.

### Characterisation of the M1FT synthesized Levan polymers

**Figure 3:**
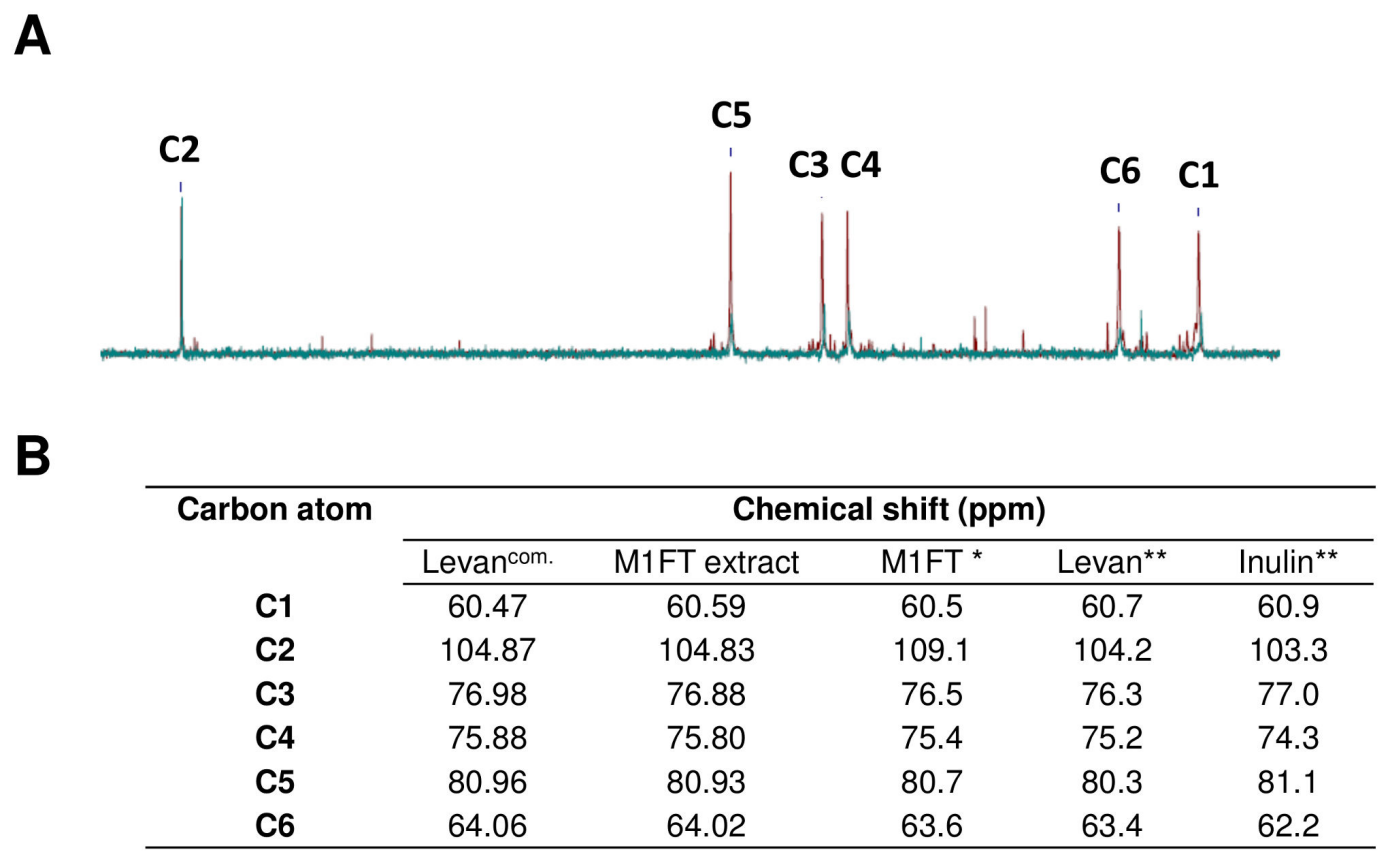
^13^C-NMR analysis of the fructan polymer produced by M1FT. (A) Overlay of the ^13^C-NMR spectra from the M1FT produced polymer and a commercially available levan polymer from *Zymomonas mobilis*. (B) Comparison of the chemical shifts for the levan polymer from *Zymomonas mobilis* (Levan^com.^); M1FT, the polymer produced by M1FT in this study; M1FT*, previously described by Kang and colleagues [24]; Levan** and Inulin**, previously described by Shimamura and colleagues [20].

The enzymatic function and also characterisation of the polymer produced by M1FT has been extensively described by Kang and colleagues [25]. It was, however, of importance to confirm whether the heterologously expressed M1FT gene does indeed produce fructan polymers when expressed in *S. cerevisiae*. The ^1^H-NMR spectrum of the purified polymer aligned to that of the commercial levan from *Zymomonas mobilis*, which was used for comparison (data not shown). The ^13^C-NMR analysis revealed the six major resonances that are associated with the β-2,1 and β-2,6 linkages of fructan polymers for both the extracted M1FT product and also the commercial levan that was included as control (Figure 3A). The ^13^C-NMR spectra also correlates with what has been previously reported for levan, with an anomeric peak (C2) at ~104 ppm and the three ring carbon (C3, C4 and C5) grouping closer together as in the case of inulin (Figure 3B). The degree of polymerization of the purified polymer (DP) was calculated to be an average of 280 fructose units.

### Heterologously expressed sucrose synthase (SuSy) has limited affinity for fructose 6-phosphate and does not produce sucrose during fermentative metabolism

The expression of the potato SuSy gene enables yeast to synthesize sucrose from glucose and fructose, the two main sugars available in grape must during wine fermentations. This creates the potential of accumulating sucrose and therefore also levan production by M1FT during industrial alcoholic fermentations. In principle, this would result in a redirection of carbon flux towards levan accumulation and away from ethanol formation, resulting in lower alcohol production from the same amount of starting sugars compared to an unmodified strain. Such an attribute would be desirable in the modern wine industry, due to consumer demand and also environmental change [26,27]. In this context it is of interest to establish whether the expressed SuSy would prefer fructose that originates from the media or fructose 6-phosphate, derived from glucose or fructose at entry into glycolysis as a substrate for the synthesis of sucrose.

**Figure 4:**
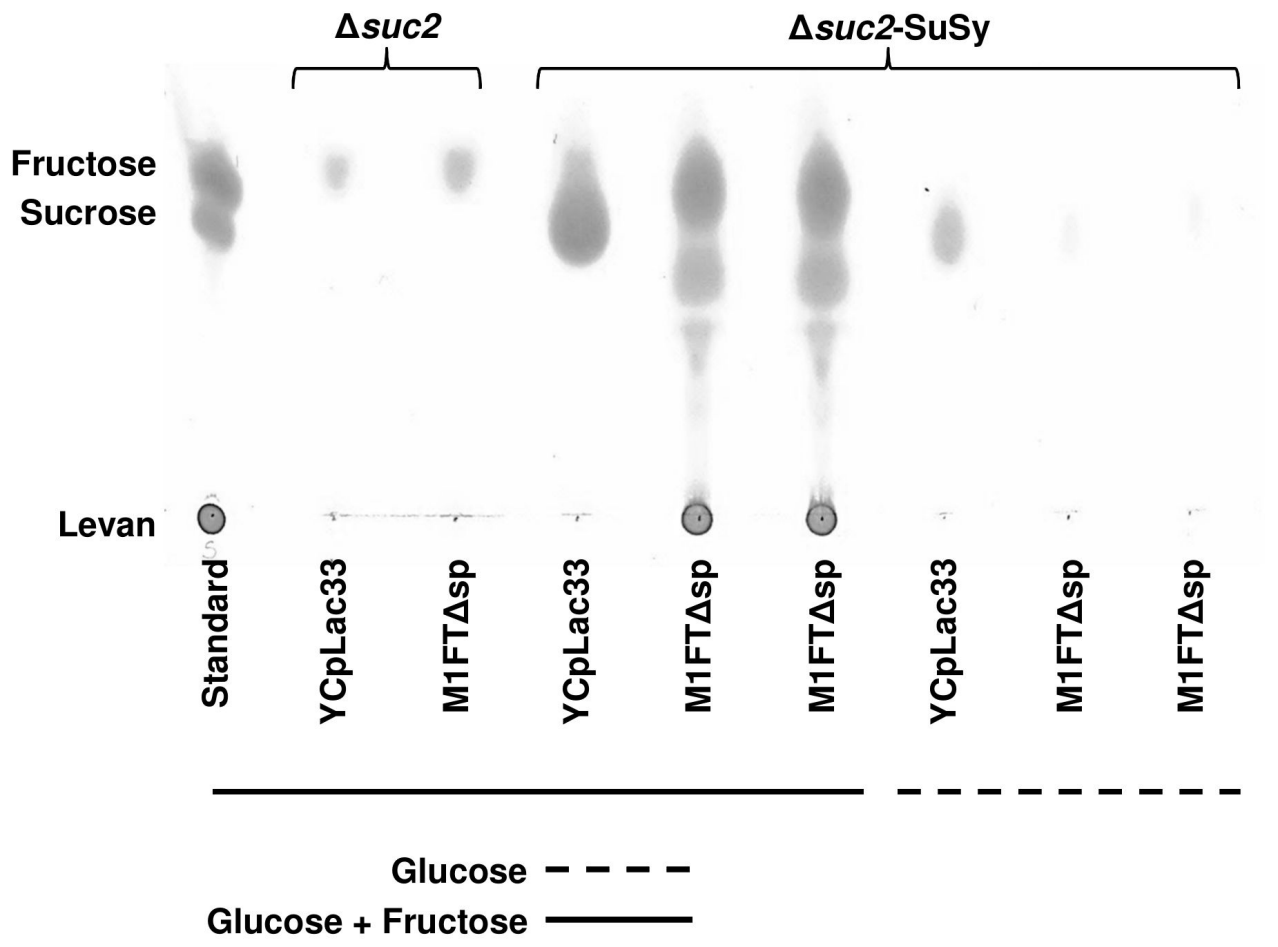
The potato sucrose synthase (SuSy) has lesser affinity for fructose 6-phosphate. Thin-layer chromatogram depicting extracts of BY4742Δ*suc2* and BY4742Δ*suc2*-SuSy strains transformed with M1FTΔsp and grown in minimal media containing either glucose or glucose and fructose to assess whether fructose 6-phosphate, originating from glucose can be used as substrate by SuSy. Strains grown in glucose and fructose are underlined with a solid line and strains grown in glucose only media with a dashed line. The decrease in sucrose formation in BY4742Δ*suc2*-SuSy grown in glucose, compared to glucose and fructose illustrates that fructose 6-phosphate, synthesized from glucose, is not a preferred substrate for SuSy. TLC plates are representative of observations confirmed by at least three biological repeats. Fructose containing molecules were visualized using a urea spray after separation on TLC plates.

To determine the source of fructose utilized by SuSy for sucrose synthesis, BY4742∆*suc2*-SuSy and BY4742∆*suc2*-SuSy-M1FTΔsp strains were cultivated in YNB media with 8% (w/v) glucose compared to the same strain grown in YNB with 4% glucose and 4% fructose as carbon sources. The glucose grown cultures will only have intracellular fructose 6-phosphate present as a product of glucose phosphorylation and isomerization. The strains were grown to saturation at 30°C, harvested and cell extracts were performed and separated on TLC as mentioned previously. A drastic reduction in sucrose production was seen in the BY4742∆*suc2*-SuSy strain when grown on glucose compared to glucose and fructose (Figure 4). From this data it can be concluded that SuSy has only a limited affinity for fructose 6-phosphate produced during glycolysis. A further decrease in the detectable amount of intracellular sucrose was noted in the BY4742∆*suc2*-SuSy-M1FT∆sp strains in comparison to the same strains without M1FT∆sp (Figure 4). This could be attributed to the sucrose hydrolytic activity of M1FT, which is expected to be dominant at lower sucrose concentrations. It has been illustrated that, at lower sucrose concentrations, enzymatic activity of levansucrases shift towards hydrolysis [28]. In addition, BY4742∆*suc2*-SuSy strains bearing the M1FT∆sp expression construct did not produce sucrose and subsequently no levan during alcoholic fermentation in the presence of 100g/L fermentable sugar (50% glucose and 50% fructose; data not shown). These strains did also not have altered ethanol yields compared to strains without SuSy (data not shown). Furthermore, the sucrose transporter (SUT) strains bearing the M1FT∆sp expression construct accumulated sucrose and also produced levan in fermentations with 100 g/L glucose supplemented with 3% sucrose, as substrate for SUT and M1FT∆sp (data not shown). These results indicate that M1FT∆sp is functional during the fermentation and that a lack of, or insufficient substrate availability causes the inability of the BY4742∆*suc2*-SuSy-M1FT∆sp strains to synthesize levan.

### Growth in rich medium results in hyper-production of levan by M1FT when combined with sucrose transporter expression

To establish if the levan production by the engineered strains can be manipulated to achieve higher yields, the various strains where grown in rich media that would support higher rates of growth, increase protein translation and in doing so elevate the potential for levan production. Specifically, media containing 1% yeast extract and 2% peptone as complete nitrogen, mineral and vitamin sources and carbon sources according to the requirements of the two different sucrose accumulation strategies were used (3% glucose and 5% sucrose for the SUT strains; 4% glucose and 4% fructose for the SuSy strains). BY4742∆*suc2* strains expressing M1FT and M1FT∆sp were used as controls and grown in both media types. The supernatants and cell extracts of cultures grown in liquid media where assessed for sucrose and levan production after two and six days respectively.

**Figure 5:**
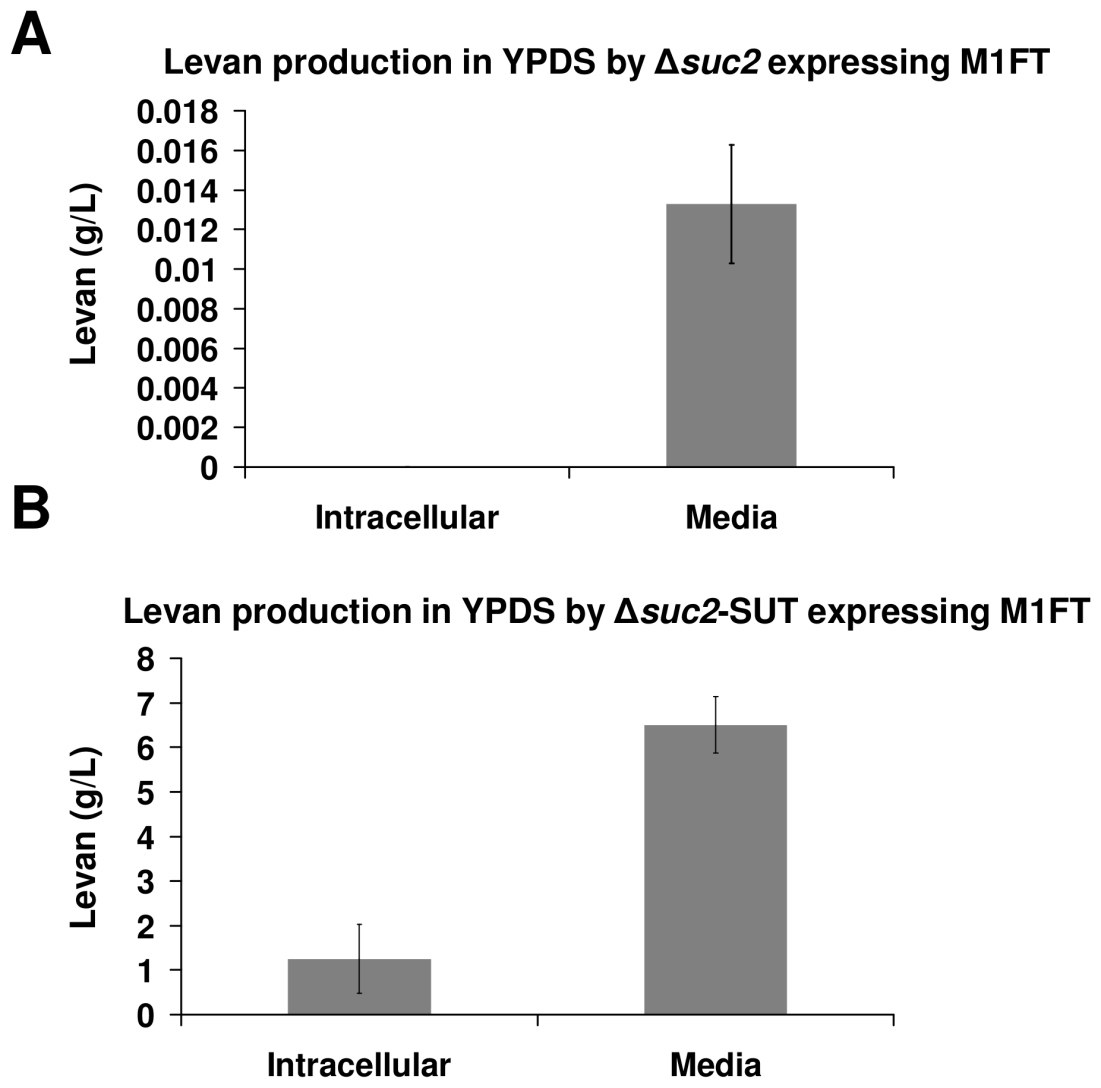
Quantification of levan production by M1FT transformed strains in rich media supplemented with sucrose (YPDS). Quantification was done by densitometric analysis of the urea stained levan polymers after visualization on TLC plates. Produced levan was either quantified from cell extracts (Intracellular) or from the growth medium (Media). Error bars indicate standard deviation (in g/L) within a triplicate sample set. (A) Levan produced by M1FT expressed in the BY4742Δ*suc2* genetic background. (B) Levan production by M1FT expressed in the BY4742Δ*suc2*-SUT genetic background.

Cultures of BY4742∆*suc2* transformed with M1FT (grown in rich media containing sucrose; YPDS) produced a small amount of levan (13.2 mg/L; SD 3.7 mg/L) in the media, detectable after six days of cultivation (Figure 5A). However, none was extractable from harvested cells or detected in M1FT∆sp expressing strains. Similar to the alcoholic fermentations, no sucrose or levan production was detectable in the cell extracts of the SuSy strains transformed with either M1FT or M1FT∆sp, when the cultures were grown in rich media. On the other hand, for the sucrose transporter (SUT) strains, sucrose accumulation was observed in the cell extracts of both M1FT and M1FT∆sp expressing cultures. Levan production in the BY4742∆*suc2*-SUT-M1FT∆sp transformed strain was comparable to that observed for the same cultures grown in minimal media (data not shown). 

**Figure 6:**
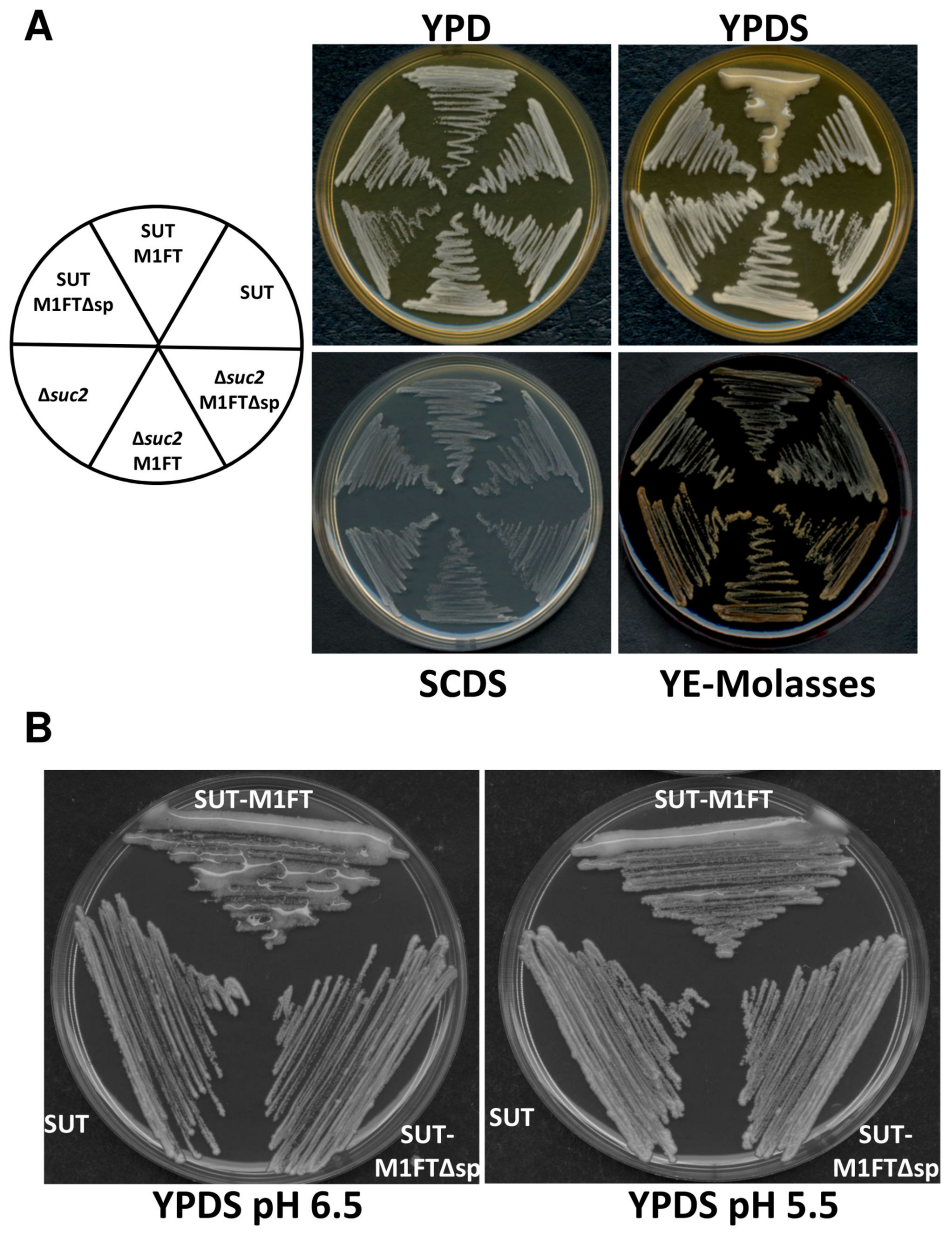
Extracellular polysaccharide (EPS) production by the M1FT expressing strains. (A) Slime production by the BY4742Δ*suc2*-SUT-M1FT yeast strain is dependent on growth medium. Cultures where grown on either rich media without sucrose (YPD), rich media with sucrose (YPDS), minimal media with sucrose (SCDS) or media containing 1% yeast extract and molasses, which was diluted to provide the same sucrose (5%) concentration as YPDS (YE-Molasses). Slime is only visible on rich media containing sucrose (YPDS). (B) The effect of pH on extracellular polymer accumulation. The production of levan as an extracellular polysaccharide (EPS) is reduced when the pH of the growth medium (YPDS) is changed from pH 6.5 (the unadjusted pH of YPDS) to pH 5.5 (which is the same as the pH of the SCDS media). Plate pictures are a representative of observations confirmed by at least three separate biological repeats.

In the case of the M1FT co-expressed with SUT, levan was produced at high levels inside the cells and also accumulated in the culture media when the cultures were grown in rich media containing 5% sucrose (YPDS; Figure 5B). In this genetic background, M1FT expression resulted in the accumulation of levan to a concentration of 6.5g/L (SD 0.63 g/L levan) in the growth medium, with a further 1.24 g/L (SD 0.7 g/L levan) extractable from the cultured cells. Together, a total yield of 15.5% levan was produced from the 50 g/L sucrose that was supplied in the media. Similar to what was observed in liquid media, the BY4742∆*suc2*-SUT-M1FT cultures accumulated extracellular levan polymers as a slime when grown on solid YPDS media (Figure 6A). No slime production was observed in the control media, which does not contain any sucrose (YPD). This phenotype is highly dependent on the growth medium on which the cultures where grown. In fact, levan accumulation was only observed on rich media containing sucrose (YPDS) and not in minimal media containing the same amount of sucrose (SCDS) or rich media containing yeast extract as nitrogen source and molasses diluted to a sucrose concentration of 5% (YE-Molasses). The most obvious difference between the various media is the variance in pH; with the pH of YPDS being 6.5 and the pH for SCDS and the molasses media slightly lower at ~5.5. Lowering the pH of YPDS to pH 5.5 (the same as SCDS) does indeed result in the production of visibly less levan than on pH 6.5 (Figure 6B) and small amounts on SCDS media at pH 6.5 (data not shown). It is, however, not currently clear what factors underlie the increase in levan production in the BY4742∆*suc2*-SUT-M1FT when grown in YPDS media compared to all other strains expressing M1FT. Future work will aim at unravelling the specific molecular interactions and possible changes that result in these observations.

## Discussion

This study set out to establish conditions that would result in the production of the fructose based polysaccharide levan, either in the growth medium or inside the cells of the yeast *S. cerevisiae*. Production of these polymers has been notoriously difficult to achieve in this industrial microorganism with no description of a levan producing strain of *S. cerevisiae* in the current literature. In this study, three different genetic platforms where created to assess the potential of the levansucrase, M1FT, from *Leuconostoc mesenteroides* to catalyse the synthesis of levan in yeast, using sucrose as a substrate. M1FT specifically presented an attractive target for expression, since it has recently been effectively expressed in *Pichia pastoris* [10]. The three genetic backgrounds that were constructed for M1FT expression included an invertase (Δ*suc2*) null mutant and added strategies using two different approaches to construct yeast strains with the ability to produce and accumulate intracellular sucrose. In the latter two strains, sucrose accumulation was accomplished by expressing either a potato sucrose synthase (SuSy) or a sucrose transporter (SUT) from spinach in the Δ*suc2* genetic background. These strategies where specifically selected, since prior studies have shown secretion to be a potential obstacle for levansucrases in yeast and also bacteria [10,29]. In addition, several studies using plant expression have illustrated functional intracellular expression to be consistently possible [1,6,7]. The selected sucrose accumulation strategies are also in line with the growth conditions that are encountered by industrial strains of *S. cerevisiae*. Specifically, the primary sugars available during wine fermentations are glucose and fructose. Molasses, a carbon source rich in sucrose, is commonly used as part of industrial base media to accumulate yeast biomass. 

The results indicate that the levansucrase from *L. mesenteroides*, M1FT, was indeed functional and able to catalyse levan synthesis when expressed in yeast. This presents the first report describing the successful expression of a levansucrase in the yeast *S. cerevisiae*. As a bacterial fructosyltransferase, M1FT has several distinctive characteristics that separate it from the other related enzymes. Firstly, most of the characterized levansucrases are monomeric enzymes within a molecular weight range of 46-73kDa. M1FT, on the other hand, functions as a dimer with a combined molecular weight of 103 kDa. M1FT is also uniquely able to transfer fructose units to maltose as an acceptor molecule, whereas other levansucrases use only sucrose [3,25]. Furthermore, although the M1FT gene was cloned from a Gram (+) bacteria, *Leuconostoc mesenteroides*, the gene sequence displays higher homology to the levansucrases from Gram (-) bacteria, with only 42% homology to the closely related *L. reuteri* levansucrase. A closely related levansucrase from *Leuconostoc citreum* shares homology with M1FT and also contains sequences with similarity to alternansucrases, which further highlights the chimeric nature of these proteins. The evidence supports a hypothesis that bacterial levansucrase genes originated through horizontal gene transfer and gene duplication events [3]. In the case of M1FT, it appears if these evolutionary changes have ironed out some of the idiosyncrasies that would be considered species specific and rendered the protein more disposed to expression across species’ boundaries. 

Levan production by M1FT was detected in association with the yeast cells when expressed in BY4742∆*suc2* cultures grown in the presence of sucrose (Figure 2B). Removal of the signal peptide (M1FT∆sp) in the same background almost entirely abolishes the production of levan on the outside of the cell. Levan production by M1FT was also confirmed in both the sucrose accumulation strains, when cultures were grown in minimal media containing the necessary carbon source combinations. An increase in the amount of levan produced inside the cell is visible upon removal of the predicted signal sequence (M1FT∆sp). Furthermore, expression of the full-length enzyme, with the signal peptide intact, resulted in the build-up of a small amount of levan in the growth medium (Figure 5A). Together, these results indicate that the M1FT signal peptide is functional and at least partly able to direct secretion of the enzyme. However, based on the levan production observed in BY4742∆*suc2-*M1FT in sucrose media, it is conceivable that a portion of the expressed pool of M1FT remains in association with the cell membrane and, even if not effectively secreted, retains its functionality. This would be consistent with previous reports for sacB from *Bacillus subtilis*, where the signal peptide was shown to impede the function of the expressed enzyme [8,29]. It would be of interest in the future to determine the location and cellular distribution of M1FT. Also, studies to direct the secretion of the enzyme using different secretion signals will establish if the presence and activity of the enzyme can be further improved in the growth medium. 

However, when considering the expression of M1FT and M1FT∆sp in the BY4742∆*suc2*-SuSy background, the levan production observed in minimal media could not be repeated when cells were grown in rich media or when the strains were used to perform alcoholic fermentations at higher sugar concentrations and anaerobic conditions (100 g/L). There would be potential commercial benefit in using such a strategy to divert carbon flux away from glycolysis and ethanol formation. Such a strategy would, in principle, enable the production of wines with reduced quantities of alcohol, a commodity which is currently in demand in the current wine industry. Explanations for the absence of levan during fermentative growth could include *S. cerevisiae*’s known preference to utilise glucose at higher rates during the initial phases of alcoholic fermentation. Fructose is commonly used at slower rates and also later in the fermentation [30]. This would result in a reduced availability of fructose to be used for sucrose synthesis by SuSy. In addition, the data indicate that SuSy has a limited ability to use fructose 6-phosphate as a substrate for sucrose synthesis and needs unphosphorylated fructose in order to function optimally. Furthermore, the known inclination of *S. cerevisiae* and other Crabtree positive yeasts to increase the rate of glycolysis in the presence of higher sugar concentrations would potentially limit access of the heterologously expressed SuSy to the substrates [31]. Similarly, the higher growth rates associated with cultures grown in rich media compared to minimal media are also known to result in increases in metabolic rates, particularly through glycolysis [31]. The optimized functioning of the yeast’s metabolism might under these conditions promptly direct fructose towards glycolytic metabolism. Potential strategies to circumvent these outcomes and generate strains that are able to effectively divert sugar from glycolysis to sucrose and fructan production could include using sucrose synthases with varying affinities for fructose or fructose 6-phosphate, using fructophilic yeast strains or generating chimeric proteins between, for instance, SuSy and substrate binding domains of the yeast hexokinases or related proteins.

The majority of current studies on levansucrases aim at the production of either the enzymes or their products for commercial use [3,6]. Levan does indeed have potential commercial applicability in the global prebiotic market, similar to inulin and fructooligosaccharides. It was therefore important to assess if the levan producing strains could be manipulated to increase production. The data generated when growing the cultures in rich media, that would support higher growth and metabolic yields gave promising and also surprising results. The expression of M1FT in combination with the spinach sucrose transporter (SUT) specifically produced high yields of levan when the cultures were grown in rich media (YPDS). A total of 7.75 g/L (SD 1.39 g/L levan) levan was produced from the 50 g/L of sucrose that was present in these cultures. This certainly provides a promising foundation from which to optimise production even further. It should also be noted that this study was performed using haploid laboratory yeast as expression system in laboratory conditions and aimed more at “proof of concept” than industrial production. By optimising secretion efficiency, yeast strain selection and media conditions it would certainly be feasible to increase the yield to levels that are commercially attractive. The appeal of using *S. cerevisiae* as a commercial levan producing organism would be, similar to *B. subtilis*, the GRAS (Generally Regarded as Safe) status of the species and also the ability to grow on inexpensive and readily available carbon sources, such as molasses. In addition, yeast by itself is an established product to which value can be added by using fructan accumulation strains. This report also opens the possibility of producing certain levansucrases as a product by heterologous expression in *S. cerevisiae*, an option which was not previously available. The optimization of such production platforms will be addressed in future studies. Previous authors reported various degrees of success when using bacterial production systems, with yields between 20 and 60 g/L [32,33]. Furthermore, by using levansucrase linked to chitin beads, yields of up to 83g/L was obtained from 20% sucrose media [34]. It remains to be shown whether the high levels of production that has been reported by natural levan producers, such as *B.subtilis*, and also enzyme based systems are attainable in *S. cerevisiae.*

When grown in rich media containing sucrose (YPDS) or on plates of the same media, BY4742∆*suc2*-SUT-M1FT produced extracellular levan to the point of visible slime formation (Figure 6). This is a commonplace observation in the case of yeast and bacteria that are naturally able to synthesize similar polymers [35,36]. In many of these organisms these extracellular polysaccharides contribute to biofilm formation and are also a factor in the determination of virulence for many species [4]. In the engineered BY4742∆*suc2*-SUT-M1FT yeast strains, extracellular accumulation was shown to be highly dependent on media conditions and only visible on YPDS media. It is not currently clear by what means the expression of SUT would facilitate the increased production of levan by M1FT that is observed in these conditions. Adjustment of the pH of the YPDS media to the same as minimal media did result in an apparent reduction in the amount of slime formation (Figure 6B). The influence of pH could, however, be due to the altered function of M1FT, SUT or both. M1FT has been shown to have a pH optimum of ~ 6.5 and the activity SUT, being a proton symporter, has been shown to be dependent on media pH when expressed in yeast [12]. The activity of plant sucrose transporters, in their native contexts and also when heterologously expressed in yeast, have been shown to be regulated by through endocytotic processes that are dependent on transporter association with lipid rafts in the cell membrane [37]. Furthermore, the function of the potato sucrose transporter in yeast is regulated by post-translational modification that is redox dependent [38]. These conditions are influenced by the growth environment and likely to contribute to the function of SUT in expression system used in this study. The contribution of these factors to the phenotypic effects that are observed when co-expressing M1FT and SuSy, will be determined in future studies. In addition, it will also be of interest to determine if the heightened levan production in the SUT, M1FT combination is specific to the two genes used or could be extended to the expression of other, related sugar polymerases.
